# Fine-grained crop pest classification based on multi-scale feature fusion and mixed attention mechanisms

**DOI:** 10.3389/fpls.2025.1500571

**Published:** 2025-04-03

**Authors:** Yiheng Qian, Zhiyong Xiao, Zhaohong Deng

**Affiliations:** School of Artificial Intelligence and Computer Science, Jiangnan University, Wuxi, China

**Keywords:** crop pest classification, deep learning, attention, multi-scale feature fusion, Convolutional Neural Network

## Abstract

Pests are a major cause of crop loss globally, and accurate pest identification is crucial for effective prevention and control strategies. This paper proposes a novel deep-learning architecture for crop pest classification, addressing the limitations of existing methods that struggle with distinguishing the fine details of pests and background interference. The proposed model is designed to balance fine-grained feature extraction with deep semantic understanding, utilizing a parallel structure composed of two main components: the Feature Fusion Module (FFM) and the Mixed Attention Module (MAM). FFM focuses on extracting key fine-grained features and fusing them across multiple scales, while MAM leverages an attention mechanism to model long-range dependencies within the channel domain, further enhancing feature representation. Additionally, a Transformer block is integrated to overcome the limitations of traditional convolutional approaches in capturing global contextual information. The proposed architecture is evaluated on three benchmark datasets—IP102, D0, and Li—demonstrating its superior performance over state-of-the-art methods. The model achieves accuracies of 75.74% on IP102, 99.82% on D0, and 98.77% on Li, highlighting its robustness and effectiveness in complex crop pest recognition tasks. These results indicate that the proposed method excels in multi-scale feature fusion and long-range dependency modeling, offering a new competitive approach to pest classification in agricultural settings.

## 1 Introduction

Due to the increasing global population, ensuring an adequate supply of global crops has become a top challenge. However, pests have a major impact on the yield reduction of commercial crops. Therefore, accurate identification of pests and timely intervention are crucial to mitigate their adverse effects on crop productivity. Pest classification is a challenging task due to the complex structures of insects and the similarities between different insect species (Ayan et al., 2020). Traditionally, pests are usually classified manually, which is time-consuming and inefficient (Dawei et al., 2019). With the development of artificial intelligence, machine vision-based pest detection equipment has been initially applied in agriculture and has replaced the traditional naked eye identification to some extent (Liu and Wang, 2021).

While traditional machine learning approaches, such as Decision Trees and Support Vector Machines, have been applied to pest classification, they are hindered by several limitations. These methods often require manual feature extraction and are highly dependent on the quality and consistency of the dataset, limiting their ability to generalize across varying conditions. The advent of Convolutional Neural Networks (CNNs) has significantly advanced the state of pest classification by enabling end-to-end learning of features directly from raw images. However, CNNs primarily excel in capturing local features and are limited by their inability to capture long-range dependencies within the image, which are essential for robust classification in complex environments.

Recent efforts (Peng and Wang, 2022; Xia et al., 2023) have integrated Vision Transformers (ViTs) into pest classification tasks to overcome the limitations of CNNs, benefiting from their self-attention mechanism to capture global contextual information. While these hybrid models have shown promise, they still face critical challenges when deployed in real-world agricultural settings: (1) In complex field environments, pest images obtained are often accompanied by high-level interference, such as uneven lighting, high similarity between pest colors and background colors, and partial occlusion of pest bodies. Therefore, an effective method is needed to suppress interference features. (2) Many pests have small body sizes and are very similar between different species, making it difficult to distinguish them. The spatial resolution size of feature maps in deep CNN architecture is limited. The details required for the precise identification of pests may be lost or become difficult to recognize at lower resolutions. Furthermore, traditional hybrid architectures often rely on the serial integration of CNN and Transformer blocks, which can result in the loss of fine-grained details during the downsampling process, especially for small pests.

Due to these practical issues, researchers have spent a lot of effort addressing the practical needs of pest detection in complex scenarios. Liu and Wang (2024) proposed a multimodal detection framework for pest targets with small proportions, diverse shapes and sizes, complex imaging backgrounds, and similarity to the background. This method can effectively distinguish subtle local differences between similar objects, thereby achieving fine-grained mapping from language to vision in complex scenes. The integration of multiple modes helps solve real-world challenges such as occlusion, low lighting, and cluttered backgrounds. However, the reliance on multimodal data collection poses potential challenges in data synchronization, which may complicate the deployment of the system in large-scale agricultural environments. In addition, the cost of data annotation needs to be considered.

Motivated by these challenges, this paper presents a novel deep-learning architecture designed to address these gaps by integrating the advantages of both CNNs and Transformers through a parallel structure. The proposed model introduces two key components: the Feature Fusion Module (FFM) and the Mixed Attention Module (MAM). FFM adaptively filters key features and fuses multi-scale information to preserve fine-grained details, while MAM builds long-range dependencies in the channel domain through a self-attention mechanism, effectively enhancing the model’s ability to capture high-level semantic features. By incorporating these components, the proposed architecture not only prevents the loss of fine-grained features during downsampling but also improves the model’s ability to suppress interference and background noise. The main contributions of this paper are summarized as follows:

1. The proposed Feature Fusion Module helps capture fine-grained features and perform multi-scale feature fusion and addresses the challenges of fine-grained feature loss during downsampling.
2. The proposed Mixed Attention Module helps build long-range dependencies in the channel domain via self-attention mechanisms and addresses the challenges of interference from complex backgrounds.
3. The proposed deep-learning architecture that integrates the strengths of both CNNs and Transformers includes two key modules and allows for the preservation of fine-grained details while also capturing deep semantic features, improving pest detection accuracy in complex field environments.

The rest of the paper is organized as follows. Section 2 reviews relevant literature on pest classification and deep learning. Section 3 describes the implementation of the proposed multi-scale feature fusion architecture. Section 4 discusses the experimental results. Section 5 discusses the limitations, applications, and future directions of the proposed method. Section 6 concludes this paper with a summary.

## 2 Related works

### 2.1 Research developments on pest classification

In the field of pest classification in the past two years, mainstream research has proposed CNN based methods (Nanni et al., 2022; Bollis et al., 2022; Zhang et al., 2022; Peng et al., 2023a; Ayan, 2023; Chen et al., 2023), Transformer-based methods (Liu et al., 2022a; Guo et al., 2023; Hechen et al., 2024), and methods based on a combination of CNN and Transformer (Peng and Wang, 2022; Xia et al., 2023). Nanni et al. (2022) proposed two new adam algorithms for deep network optimization based on DGrad that introduce a scaling factor in the learning rate. Bollis et al. (2022) proposed an attention-based activation map approach developed to improve the classification of tiny regions called two-weighted activation mapping, which produces locations using feature map scores learned from class labels. Zhang et al. (2022) proposed a low-energy consumption hybrid ResNet structure to reduce the energy burden of network computing. They presented an optimal AM-ResNet design method through a detailed experimental analysis of the performance differences between building blocks in two typical ResNet variants, ResNet-20 and ResNet-32. Peng et al. (2023a) built a pest dataset named HQIP102 and proposed a pest identification model named MADN based on DenseNet. Ayan (2023) proposed a new hyperparameter optimization strategy based on a genetic algorithm for pre-trained CNN models in pest classification. The proposed method was tested with three CNN models (MobileNet-V2, DenseNet-121, and InceptionResNet-V2). Chen et al. (2023) proposed a multi-image fusion recognition method to perform fusion recognition on multiple images of the same pest instead of the conventional single image. Liu et al. (2022a) proposed a self-supervised Transformer-based pre-training method using latent semantic masking auto-encoder (LSMAE) and a feature relationship conditional filtering (FRCF) based on k-NN graph to enhance global information interaction and discriminative feature representation in models. Guo et al. (2023) considered the class ambiguity problem and converted the conventional one-label pest classification task into a multi label one. Hechen et al. (2024) established a novel Dilated-Windows-based Vision Transformer with Efficient-Suppressive-self-attention (DWViT-ES) architecture to confine the range of self-attention within a local region to reduce the computation complexity. Peng and Wang (2022) designed the Transformer as the classification head of CNN to balance accuracy and efficiency. Xia et al. (2023) proposed a new pest classification method based on a DenseNet and an improved Vision Transformer in response to the problems of low efficiency and inadaptability to the large-scale environment of existing pest classification methods. CNN has the advantages of strong local perception, robustness, and scalability, but it also has some limitations. For example, the lack of perception of global information leads to poor processing of long sequences; CNN uses convolutional operations to extract features, which can result in the loss of positional information in the input data. Transformer can capture long-distance dependencies through its self-attention mechanism and has excellent global modeling ability. However, its limitations are as follows: due to the lack of inductive bias characteristics similar to CNN, Transformer lacks sufficient generalization ability in the case of insufficient data, making it difficult to achieve ideal results; Due to the high computational complexity of the attention mechanism and the fact that its computational rate is the second power of the increase in image size, it requires very high hardware requirements; Lack of spatial induction bias and insensitivity to spatial information. **Table 1** illustrates the differences between the various studies. Compared with other studies, the contribution of this paper is to propose a hybrid architecture and introduce multi-scale information of pests into the architecture.

**Table 1:** Different studies on pest classification.

Study	Architecture	Single-scale or Multi-scale
Ren et al. (2019)	CNN	Single-scale
Nanni et al. (2020)	CNN	Single-scale
Ayan et al. (2020)	CNN	Single-scale
Yang et al. (2021)	CNN	Single-scale
Nanni et al. (2022)	CNN	Single-scale
Zhang et al. (2022)	CNN	Single-scale
Ayan (2023)	CNN	Single-scale
Chen et al. (2023)	CNN	Single-scale
Guo et al. (2023)	Transformer	Single-scale
Hechen et al. (2024)	Transformer	Single-scale
Peng and Wang (2022)	CNN & Transformer	Single-scale
Xia et al. (2023)	CNN & Transformer	Single-scale
Ours	CNN & Transformer	Multi-scale

### 2.2 Hybrid of CNN and transformer

Transformer was originally proposed as a sequence-to-sequence model for machine translation (Lin et al., 2022). Transformer has not only shined in the field of NLP but has also achieved outstanding performance in areas such as computer vision. Research has shown that Transformer-based pre-trained models can achieve state-of-the-art in most computer vision tasks (He et al., 2023). In recent years, Transformer architecture has become a research hotspot in computer vision due to its outstanding performance. The Vision-Transformer (Dosovitskiy et al., 2020) has been proven to outperform the best CNN classification networks (Han et al., 2023). However, its weaker inductive bias is generally found to cause an increased reliance on model regularization or data augmentation when applied to smaller datasets (Steiner et al., 2021), which means that the pure ViT network is not the best choice when applied to a specialized smaller dataset. Due to the significant covariance within the local neighborhood and the gradual stabilization of the image, CNN can effectively process the image with the help of bias. However, strong bias also limits CNN when sufficient data is available (Liu et al., 2023). Therefore, many studies have attempted to introduce the locality of CNN into Transformers and have achieved good results (D’Ascoli et al., 2021; Yuan et al., 2021; Li et al., 2021; Xiao et al., 2021; Dai et al., 2021; Xiao et al., 2022; Peng et al., 2023b; Gao et al., 2024; Xiao et al., 2024; Ji et al., 2023), demonstrating the effectiveness of combining CNNs and Transformers.

## 3 Materials and methods

### 3.1 Data collection

**Figure 1:**
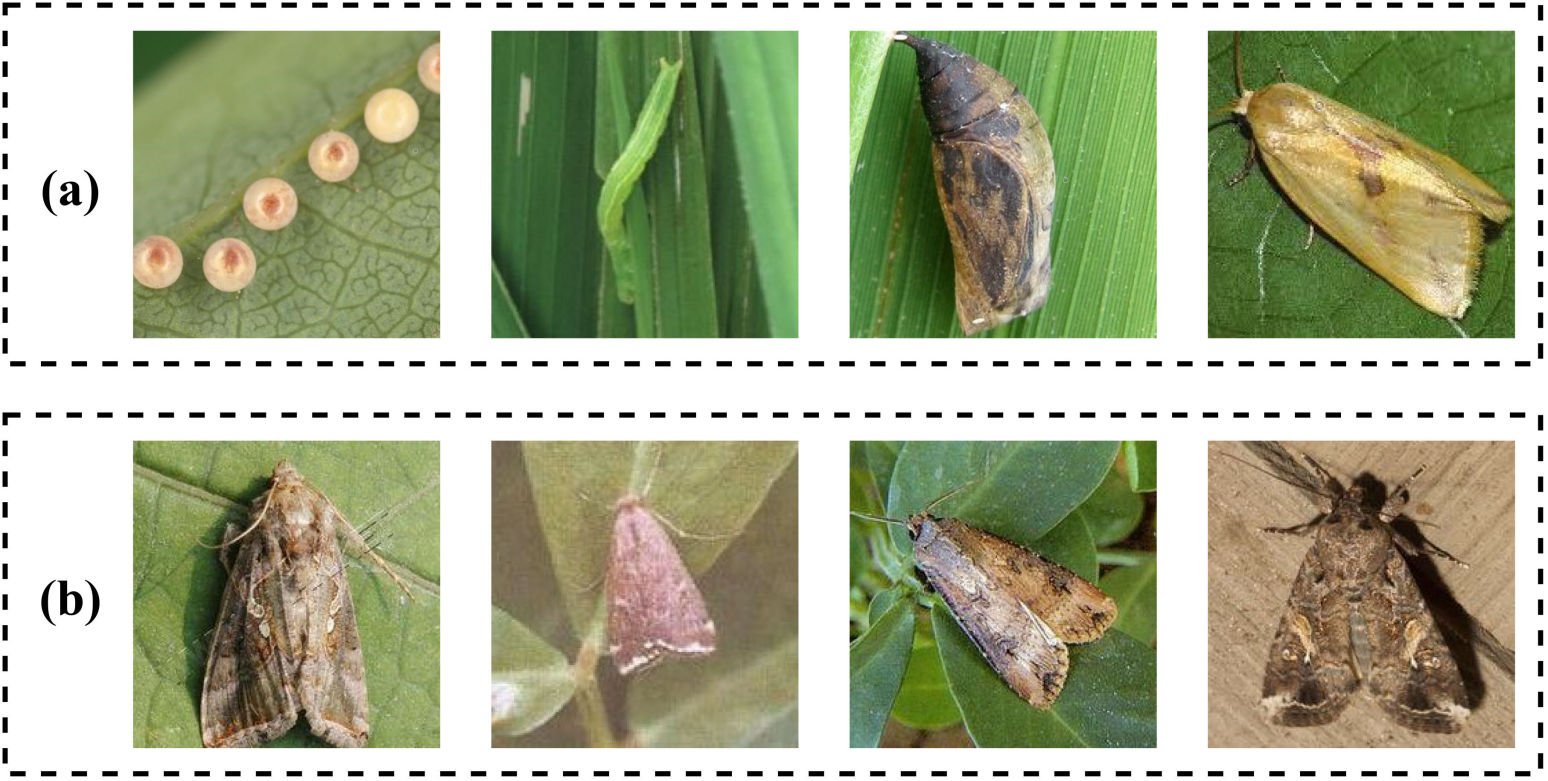
Samples in IP102 dataset. **(a)** Different forms of Naranga aenescens Moore that contain eggs, larvae, pupa, and adults. **(b)** Adults of different types of pests, which are difficult to distinguish.

The first insect dataset in the experiment is collected from the Insect Pest dataset (IP102) (Wu et al., 2019), which includes 75222 images and 102 classes of crop insect pests. In a dataset, the imbalance in the number of samples between different categories often leads to classifiers tending toward categories with more samples and performing poorly on categories with fewer samples. The problem of sample imbalance often occurs in data with a long-tail distribution. The degree of long tail distribution can be measured by the imbalance ratio (IR), which is the number of samples in the class with the most and divided by the number of samples in the class with the least. The larger the IR value, the higher the degree of imbalance in the dataset. IP102 contains images of pests in various environments, with varying quality, and some even contain noise and watermarks. This simulates the complex background, changing lighting conditions, and different perspectives in the real world, consistent with the natural shooting environment of farmland. Therefore, it has good representativeness. IP102 is also a large-scale pest dataset with a severe long-tail distribution and an IR value of 82. The largest class contains 3444 images, while the smallest class contains 42 images, which may lead to some potential biases. The dataset is split into 45095 images for training, 7508 for validation, and 22619 for the classification task. There are challenges when applying the dataset to practical use. Primarily, the dataset retains images covering different life cycles of insects, which can hardly be classified into the same category. **Figure 1a** shows different pests in IP102 containing egg, larva, pupa, and adult. Additionally, there are images of different species that are similar and difficult to distinguish, as shown in **Figure 1b**. Finally, samples from different categories are imbalanced.

The other two datasets (D0 (Xie et al., 2018) and Li (Li et al., 2020)) are used to test the generalization ability of the proposed model. The D0 dataset contains a total of 4508 images with 40 categories. Before the training process, the dataset is split into two groups: training and testing. Randomly, 75% of images are selected for training, and 25% are selected for testing (the reason for choosing this ratio is discussed in Section 4.6). Li is a small dataset presented in Li et al. (2020), which contains 5629 images in 10 different classes. The same splitting strategy as D0 is used to split Li.

### 3.2 The proposed model

**Figure 2:**
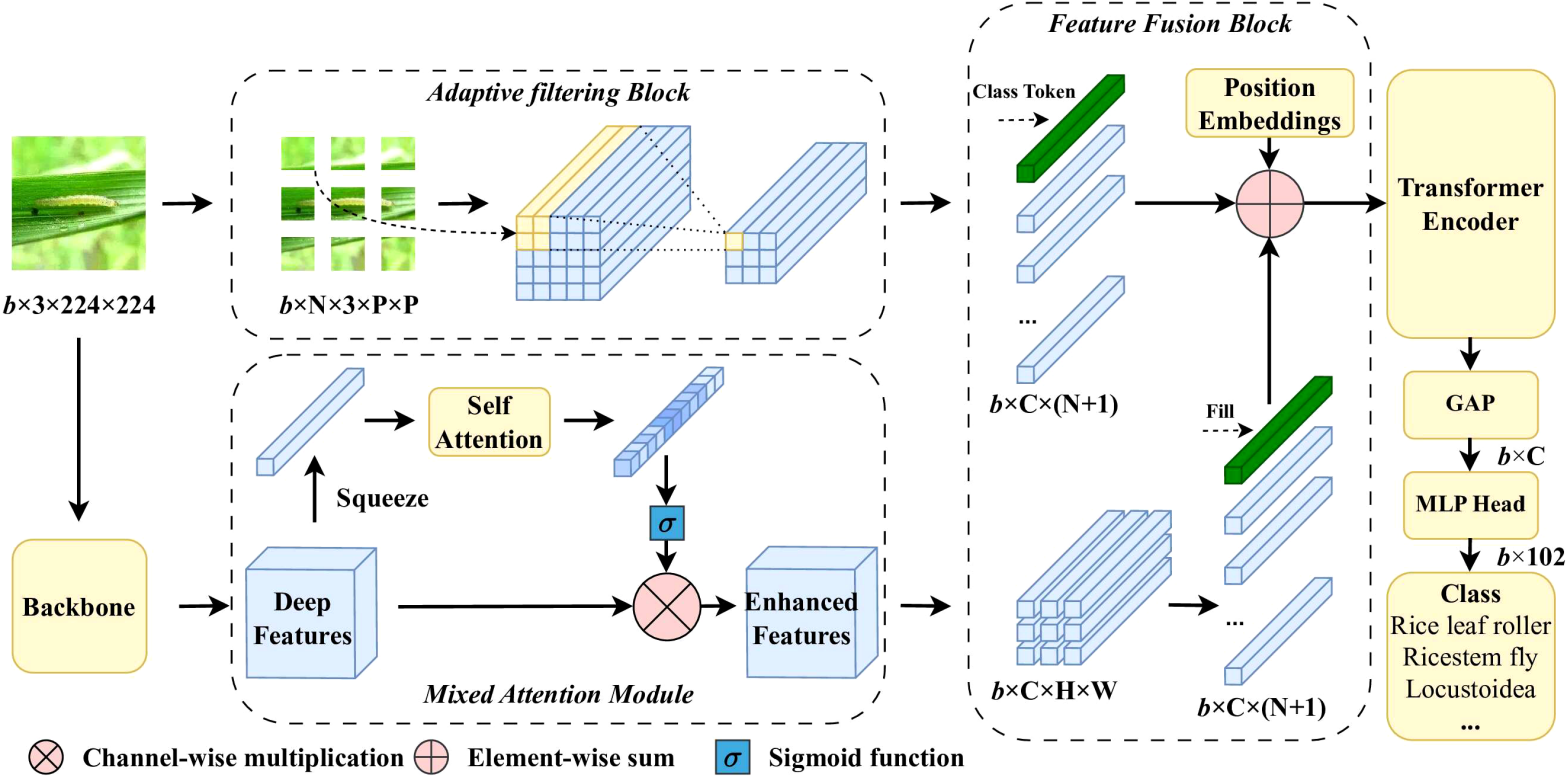
Pipeline of the proposed model.

A novel architecture is proposed to balance the ability to extract fine-grained features and local deep semantic features simultaneously while integrating advantages both from CNN and Transformer. Below is an overview of the proposed model, as shown in **Figure 2**. It consists of a feature enhancement module (MAM) and a feature fusion module (FFM). As shown in the lower part of **Figure 2**, MAM is applied to enhance the extracted deep semantic features and suppress irrelevant features. In this stage, CNN is used as the backbone network for feature extraction due to its excellent ability to perceive local features. The pooling layer and classification layer of the backbone network are removed to retain feature maps. Then, the feature maps extracted by the backbone network are processed through MAM. As shown in the upper part of **Figure 2**, FFM extracts fine-grained features and fuses features from two scales. Specifically, the original image is divided into patches, the features of which are adaptively selected to obtain important fine-grained features. The Transformer block is used after that to process these fused features. Finally, a multilayer perceptron head is used for classification. The detailed module design is presented in the following text.

#### 3.2.1 Mixed Attention Module (MAM)

The Squeeze-and-Excitation (SE) module proposed by Hu et al. (2018) was designed to reduce the interference of irrelevant features in the picture by explicitly modeling the interdependencies between the channels of its convolutional features. In this module, an input image $X\in {\mathbb{R}}^{C^{\prime }\times H^{\prime }\times W^{\prime }}$ passes through the backbone network to be transformed into a feature map $U\in {\mathbb{R}}^{C\times H\times W}$ by convolutional operations (Equations 1, 2), which refers to a transformation composed of convolutional operations. The feature map *U* can be expressed as:


(1) $$u_{c}=v_{c}*X\overset{{k=1}^{\text{C'}}}{\underset{\Sigma }{=}}v_{c}^{k}*x^{k},$$

(2) $$U=\left[u_{1},u_{2},\ldots ,u_{c}\right],$$

where *u_c_* refers to a single channel of *U*, ∗ denotes convolution, *v_c_* refers to a 3D spatial kernel, and *k* refers to the channel on which the convolutional operations are located. To explicitly model the interdependencies between the channels, global average pooling is applied to squeeze global information into a channel (Equations 3, 4), which defines the squeeze operation to be:


(3) $$z_{c}=F_{sq}\left(u_{c}\right)=\overset{{j=1}^{\text{W}}}{\underset{\Sigma }{\overset{{i=1}^{\text{H}}}{\underset{\Sigma }{\frac{1}{H\times W}}}}}u_{c}\left(i,j\right),$$

(4) $$z=\left[z_{1},z_{2},\ldots ,z_{c}\right],$$

where *z_c_* refers to the value of the channel into which *u_c_* is squeezed. After that, an excitation operation is employed to capture channel-wise dependencies fully (Equation 5):


(5) $$s=F_{ex}\left(z,W\right)=\sigma \left(W_{2}\delta \left(W_{1}z\right)\right),$$

where *δ* refers to the ReLU (Nair and Hinton, 2010) function, *σ* refers to the Sigmoid function, $W_{1}\in {\mathbb{R}}^{\frac{C}{r}\times C}$ and $W_{2}\in {\mathbb{R}}^{C\times \frac{C}{r}}$ . The excitation operation consists of two fully connected layers, where *r* refers to the reduction ratio of the channel-reduction layer. Finally, the output $\tilde{X}\in {\mathbb{R}}^{C\times H\times W}$ is calculated by scaling *U* with *s* (Equations 6–8):


(6) $$s=\left[s_{1},s_{2},\ldots ,s_{c}\right],$$

(7) $${\tilde{x}}_{c}=F_{scale}\left(u_{c},s_{c}\right)=s_{c}u_{c},$$

(8) $$\tilde{X}=\left[{\tilde{x}}_{1},{\tilde{x}}_{2},\ldots ,{\tilde{x}}_{c}\right],$$

where *s_c_* refers to one of the outputs of the fully connected layer.

The excitation operation based on fully connected layers can perform well but may suffer from parameter redundancy and lack of long-range dependency modeling. From this point of view, the excitation operation is modified to obtain a more effective implementation. The methodology is as follows:

##### 3.2.1.1 Fully-connected replace

**Figure 3:**
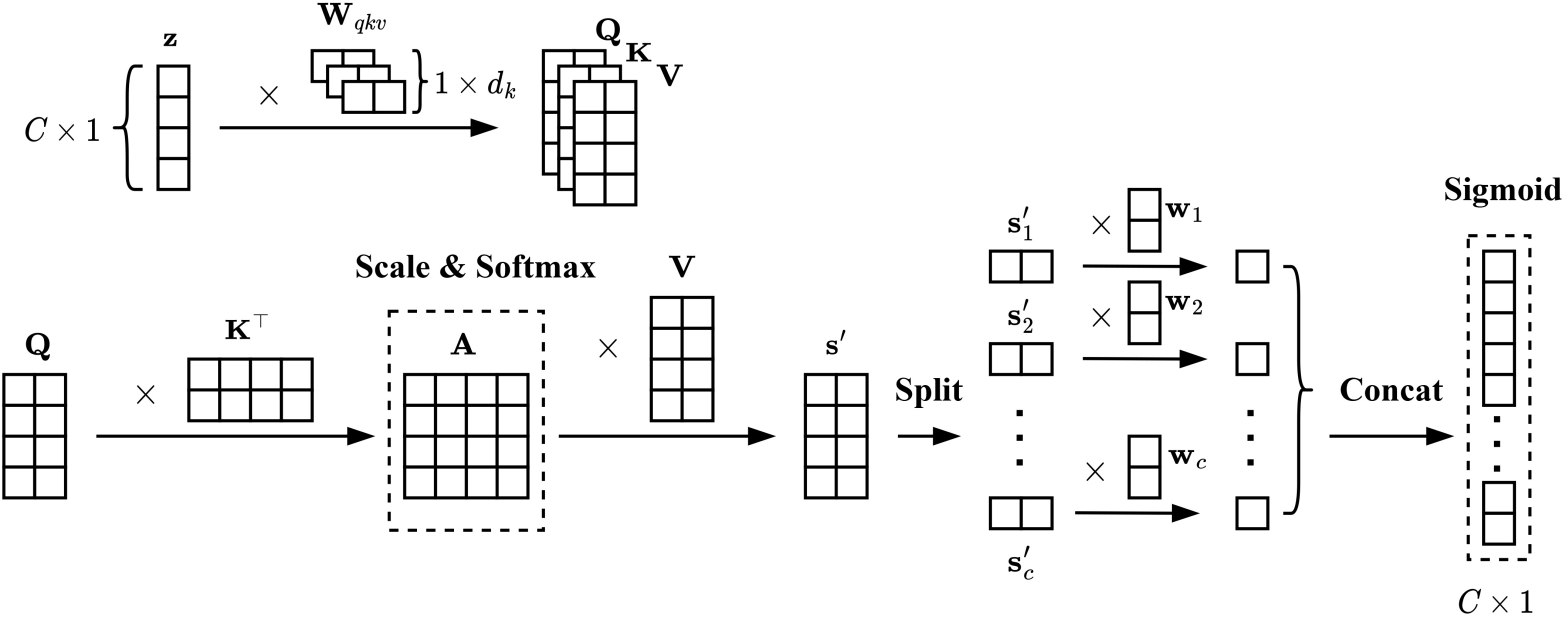
Excitation based on mixed-attention mechanism.


Self-attention mechanism is introduced to the excitation operation, which adds long-range dependencies to channels and reduces the number of parameters for the excitation operation. As shown in **Figure 3**, the input tensor $z\in {\mathbb{R}}^{C\times 1}$ is transformed into *Q*, *K*, and *V* by linear transformations $W_{q}$ , $W_{k}$ , and $W_{v}$ (Equations 9–11), which can be expressed as:

(9) $$Q=z\cdot W_{q},$$

(10) $$K=z\cdot W_{k},$$

(11) $$V=z\cdot W_{v},$$

where $W_{q}$ , $W_{k}$ and $W_{v}$ are learnable projection matrices, $W_{q},W_{k},W_{v}\in {\mathbb{R}}^{1\times d_{k}}$ and $Q,K,V\in {\mathbb{R}}^{C\times d_{k}}$ . Then, the weighted channel information $s^{\prime }$ can be calculated as (Equation 12):


(12) $$s^{\prime }=softmax\left(\frac{QK^{⊤}}{\sqrt{d_{k}}}\right)V,$$

where *d_k_* refers to the length of channel features after linear transformation (the value of this parameter is discussed in Section 4.3), that is, the number of columns of *Q*, *K*, and *V*. Finally, $s^{\prime }$ is divided into chunks, a linear transformation is then performed on each chunk, and all these chunks are concatenated to obtain the channel-wise dependencies (Equation 13–15), which can be expressed as:


(13) $$s^{\prime }=\left[s_{1}^{\prime },s_{2}^{\prime },\ldots ,s_{c}^{\prime }\right],$$

(14) $$s_{c}=\sigma \left(s_{c}^{\prime }w_{c}\right),$$

(15) $$s=\left[s_{1},s_{2},\ldots ,s_{c}\right],$$

where $s_{c}^{\prime }$ refers to one chunk, *w_c_* refers to the weights of a linear transformation for a specific chunk, and *σ* refers to the Sigmoid function. This module aims to build long-range dependencies in the channel domain and balance accuracy and model parameters at the same time.

##### 3.2.1.2 Discussion

The Mixed Attention Module (MAM) builds upon the foundation laid by the Squeeze-and Excitation (SE) module proposed by Hu et al. (2018), which focuses on modeling the interdependencies between the channels of convolutional feature maps. The SE module employs a channel-wise excitation mechanism via fully connected layers, effectively re-weighting each channel based on its global average pooled features. While this method has proven successful in improving model performance, it has limitations that hinder its potential in certain applications. Specifically, the fully connected layers used in the excitation operation are prone to parameter redundancy and fail to capture long-range dependencies within the channels, which may be crucial for certain tasks, especially in high-dimensional feature spaces. To address these limitations, the MAM introduces a novel approach by replacing the traditional fully connected excitation mechanism with a self-attention mechanism. Self-attention has the distinct advantage of capturing long-range dependencies within the input feature map by considering relationships between all channels, irrespective of their spatial locations. This introduces more flexible modeling of channel dependencies, enabling the module to learn more complex, global relationships between channels compared to the local, pairwise relationships modeled by the fully connected layers in the SE module. In later experiments, the effectiveness of MAM will be verified.

#### 3.2.2 Feature Fusion Module (FFM)

**Figure 4:**
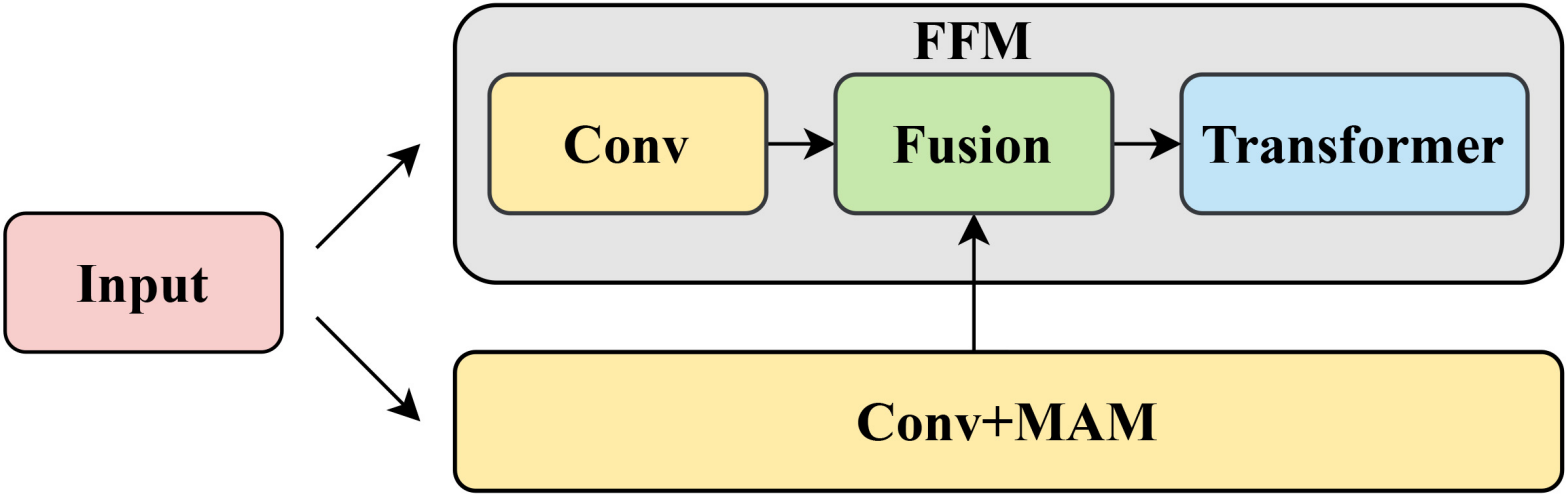
Information flow between CNN, Transformer, and FFM.

The proposed FFM consists of three components: adaptive filtering block, feature fusion block, and Transformer block. **Figure 4** shows the information flow between CNN, Transformer, and FFM.

The function of the adaptive filtering block is to select key image patches and filter out redundant information. As shown in **Figure 2**. The input image $x\in {\mathbb{R}}^{3\times 224\times 224}$ is divided into patches, representing different image regions. Since pests only occupy a small portion of space, only a few important regions need to be focused on. As a result, a projection is then performed on these patches so that the model can learn which region plays a crucial role in classification. Then, position embeddings are added to the patches to fully utilize the images’ contextual and positional information (Equations 16, 17). The process can be expressed as:


(16) $$z=\left[x_{class};x_{p}^{1}E;x_{p}^{2}E;\ldots ;x_{p}^{N}E\right]+E_{pos},$$

(17) $$x_{p}\in {\mathbb{R}}^{N\times 3\times P\times P},E\in {\mathbb{R}}^{N\times 3\times P\times P},E_{pos}\in {\mathbb{R}}^{C\times \left(N+1\right)},$$

where *x_p_* refers to one patch of the image, *x_class_* refers to the class token which is appended to the patches as a specific feature for classification, *E* is a convolutional kernel, *E_pos_* are learnable 1D position embeddings, *C* refers to the number of channels, *P* refers to the width of a patch, and *N* refers to the number of patches. It is worth noting that a convolution layer is used for projection instead of a linear one to avoid parameter redundancy in the practical operation.

The function of the feature fusion block is to fuse the fine-grained features *z* and deep semantic features $x^{\prime }$ , which are obtained by the other branch of the proposed model. To achieve this, a feature alignment strategy is designed as the bridge. To align the channel dimensions, FFM reshapes the feature maps and concatenates a zero vector to the reshaped feature maps in the corresponding position of the class token. The fused features can be calculated as (Equations 18):


(18) $$z_{0}=\left[0;reshape\left(x^{\prime }\right)\right]+z$$

The function of the Transformer block is to integrate the concepts of locality, inductive bias, and shift invariance from CNN and overcome the inherent limitations of traditional convolution. In our implementation, the fused features *z*_0_ are sent into the Transformer block. The Transformer block comprises a multi-head self-attention (MSA) block and an MLP block, both of which are normalized using LayerNorm (LN). For an input sequence *z*_0_, the self-attention mechanism calculates the weighted sum of each element in the sequence. Multi-head self-attention is a combination of several self-attention operations, which need to run *h* self-attention mechanisms in parallel. Then, these outputs are concatenated and transformed into the final output (Equation 19):


(19) $$MSA\left(z\right)=concat\left(A_{1}\left(z\right),A_{2}\left(z\right),\ldots ,A_{h}\left(z\right)\right)W_{msa},$$

where *A_h_* refers to the self-attention operation, *W_msa_* refers to the weight matrix of linear transformation. The process of the Transformer can be expressed as (Equations 20–22):


(20) $$z_{l}^{'}=MSA\left(LN\left(z_{l-1}\right)\right)+z_{l-1},\;l=1,2,\ldots ,L,$$

(21) $$z_{l}=MLP\left(LN\left(z_{l}^{'}\right)\right)+z_{l}^{'},\;l=1,2,\ldots ,L,$$

(22) $$y=LN\left(z_{L}\right),$$

where *z_l_* refers to the output of the *l*th block, *L* refers to the depth of the Transformer block.

##### 3.2.2.1 Discussion

The design of FFM provides a balanced approach by focusing on fine-grained feature extraction, multi-scale fusion, and long-range dependency modeling, thereby overcoming the limitations of traditional CNN architectures. The position Embeddings allow the model to retain spatial relationships between different regions of the image, which is crucial for pest classification, as the spatial arrangement of pests can significantly impact their identification. These improvements make the model more robust to disturbances, more capable of handling small pests, and more efficient in feature extraction, all of which are crucial for accurately classifying pests in complex agricultural environments. The effectiveness of this module will be verified in later experiments.

### 3.3 Implementation details

In the proposed model, several design choices regarding the input tensor dimensions, convolutional kernel size, stride, and activation functions play a critical role in ensuring the module’s effectiveness and computational efficiency.

#### 3.3.1 Input Tensor Dimensions

The input image is represented as a tensor $x\in {\mathbb{R}}^{3\times 224\times 224}$ , where 3 denotes the color channels (RGB) and 224 × 224 is the spatial resolution of the image. This resolution strikes a balance between capturing sufficient detail in pest images and ensuring computational efficiency. Given that pests occupy only a small portion of the image, a resolution of 224×224 allows the network to capture the necessary fine-grained features without incurring excessive computational costs.

#### 3.3.2 Convolutional Kernel Size and Stride

In the adaptive filtering block, a convolution layer is employed for patch projection with a kernel size of 32. The stride is set to 32. This ensures that spatial details important for pest identification are retained during the patch extraction process. Furthermore, the use of a convolution kernel instead of a fully connected layer avoids parameter redundancy and enhances computational efficiency.

#### 3.3.3 Activation Functions

MIM employs the Sigmoid activation function in the output of the excitation operation. Sigmoid, defined as $\sigma \left(x\right)=\frac{1}{1+e^{-x}}$ , is particularly well-suited for scenarios where the output needs to be interpreted as probabilities or to emphasize the relevance of individual features in a range between 0 and 1. In MAM, Sigmoid operates on the attention scores to generate a soft attention map, allowing the model to weigh the importance of different channels adaptively. This probabilistic interpretation enables the network to focus more on informative parts of the input, enhancing the feature extraction process while suppressing irrelevant or noisy features.

## 4 Results

### 4.1 Experimental setup

#### 4.1.1 Evaluation metrics

Considering that the quantities of different types of pests are unbalanced in IP102, D0, and Li, two evaluation metrics are chosen, namely accuracy and weighted-F1, where accuracy is the ratio of the correctly classified samples to the total number of samples, and weighted-F1 is a weighted average of F1 for each category, to measure the precision and recall of a classification model. The evaluation metrics are defined as follows (Equations 23–27):


(23) $$Precision_{i}=\frac{TP_{i}}{TP_{i}+FP_{i}},$$

(24) $$Recall_{i}=\frac{TP_{i}}{TP_{i}+FN_{i}},$$

(25) $$F1_{i}=2\cdot \frac{Precision_{i}\cdot Recall_{i}}{Precision_{i}+Recall_{i}},$$

(26) $$F1_{weighted}\overset{{i=1}^{\text{n}}}{\underset{\Sigma }{=}}w_{i}\cdot F1_{i},$$

(27) $$Accuracy=\frac{TP+TN}{TP+TN+FP+FN},$$

where *i* refers to a specific category, *TP_i_* refers to the number of samples correctly classified as *i*. *FP_i_* refers to the number of samples incorrectly classified into other categories. *FN_i_* refers to the number of samples that belong to other categories but are classified as *i*. *TN_i_* refers to the number of samples that belong to other categories and are not classified as *i*, and *w_i_* refers to the proportion of samples that belong to *i*.

#### 4.1.2 Hyperparameter settings

All modules for ablation analysis and comparison experiments are implemented with the Pytorch library and trained on one GeForce RTX 2080Ti GPU. All the parameters of the proposed are randomly initialized except the backbone network. Standard practices are followed, and data augmentation is performed with random cropping using the scale to a size of 224 × 224 pixels. To avoid overfitting, dropout (Srivastava et al., 2014) is employed in the model and set to 0.1. AdamW is used as an optimizer when fine-tuning the model. The batch size is set to 32, and the learning rate is set to 5 × 10^−5^, the same linear scaling rule (*lr* × *batchsize/*256) is used as Goyal et al. (2017) with base *lr* = 0.0004. The model is trained for 150 epochs on IP102 and 10 epochs on D0 and Li. The hidden dimensions *d_k_* in Equation 12 are set to 384 by default.

### 4.2 Backbone networks

To select a suitable CNN as the backbone network and balance accuracy and inference time for different model configurations, experiments are conducted using several mainstream CNNs combined with the proposed method. The experimental results are shown in **Table 2**. All the models are trained, validated, and tested on IP102 with an input image resolution of 224 × 224. As seen in the table, using ConvNext-L as the backbone network of the model has the best classification performance. Therefore, it is used as the backbone network of the model.

**Table 2:** Classification performance achieved by using different CNNs as the backbone network of the proposed model.

Backbone Network	Params (M)	Inference Time (ms)	Accuracy (%)
EfficientNet-B0	27.754	18.68	70.59
VGG-16	19.496	17.99	67.69
VGG-19	24.806	18.07	68.30
ResNet-50	80.736	18.89	67.70
ResNet-101	99.368	19.92	67.66
ResNet-152	115.011	19.38	67.82
ConvNeXt-S	58.970	18.34	74.29
ConvNeXt-B	103.399	18.94	74.51
ConvNeXt-L	224.694	21.72	75.74

### 4.3 Effectiveness of MAM

The value of *d_k_*: the hyperparameter *d_k_* introduced in Equation 12 affects the complexity and prediction accuracy of the model. To investigate the impact of this hyperparameter and to determine its value, MAM is integrated into the backbone network, and *d_k_* is set to a range of different values. As shown in **Table 3**, setting *d_k_* = 384 achieves a good balance between accuracy and complexity, while simply increasing model complexity does not improve classification accuracy.

**Table 3:** Accuracy (%) on IP102 and parameter sizes for integrating the SE module and MAM into the backbone network respectively with different values of hyperparameters.

Method	Hyperparameter	Params (M)	Accuracy (%)
SE	*r* = 16	196.655	75.26
	*r* = 8	196.950	75.02
	*r* = 4	197.540	75.24
	*r* = 2	198.719	75.08
MAM	*d_k_* = 81	196.361	74.95
	*d_k_* = 192	196.361	75.05
	***d_k_* =384**	**196.363**	**75.41**
	*d_k_* = 768	196.365	74.97
ConvNeXt-L	–	196.360	74.78

The bold values are the results obtained by our method, to emphasize.

MAM aims to build long-range dependencies on channels so that the model can learn more discriminative implicit features. Also, it must offer a good balance between performance and model complexity for practical use. To validate the effectiveness of the proposed module, the SE module and MAM are integrated into the backbone network, respectively, with the reduction ratio *r* set to a range of different values for a more intuitive comparison. The comparison result in **Table 3** shows that setting *d_k_* = 384 in MAM achieves the highest accuracy, higher than setting *r* = 16 in the SE module, but with fewer parameters.

### 4.4 Effectiveness of FFM

**Figure 5:**
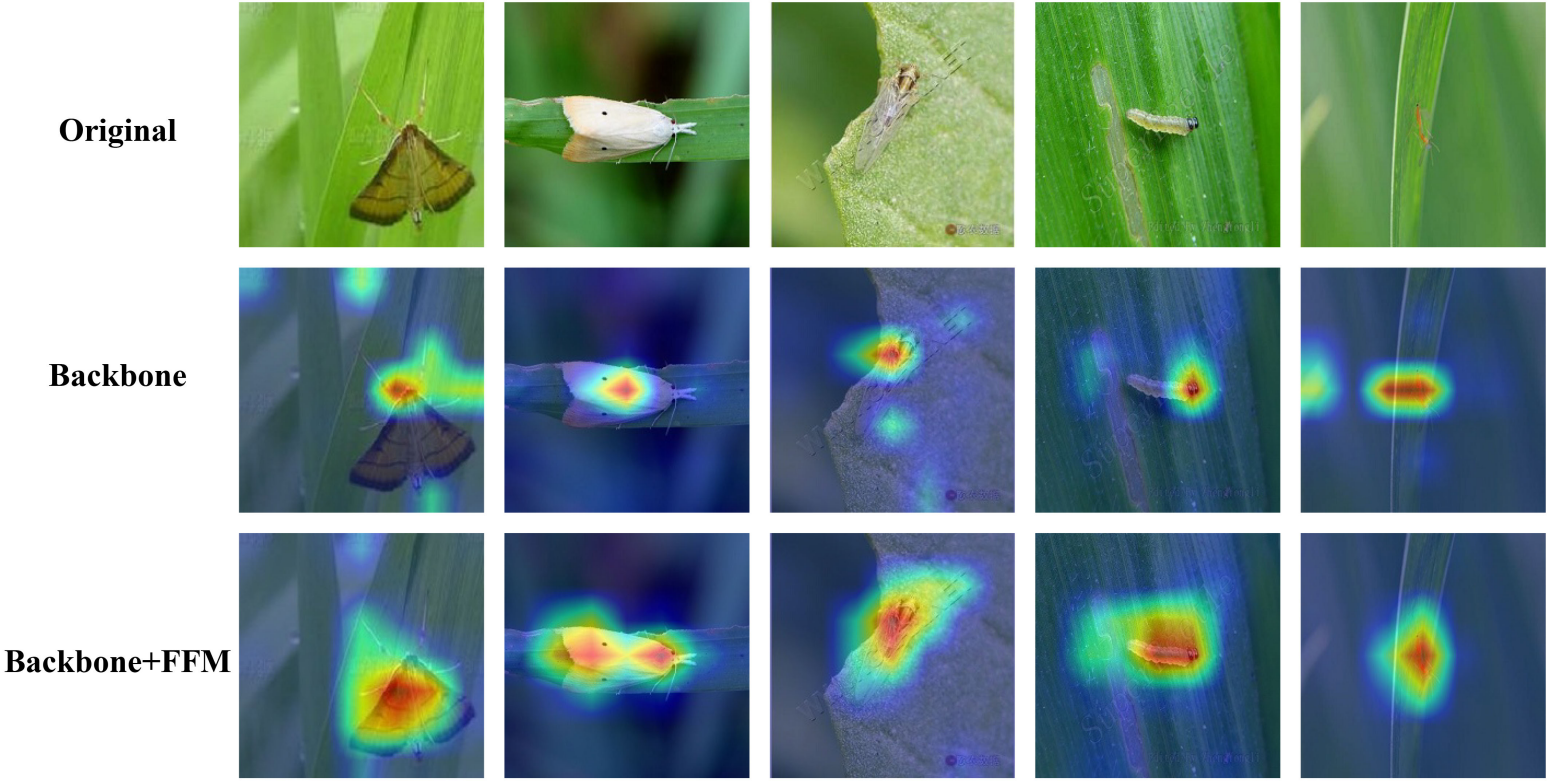
Images of the Grad-CAM generated by each network. The first row of images consists of 5 original images of different pests. The images in the second row are the GCAM images obtained by the backbone network. The images in the third row are the GCAM images obtained by the feature fusion Module. Through the feature fusion module, the model can more comprehensively extract the features of pests.

In this paper, FFM is designed to utilize fine-grained information from pest images to accurately classify pests with small body sizes or similar shapes and colors. To further evaluate the effectiveness of the proposed method, the method of Grad-CAM (Gradient-weighted Class Activation Mapping) (Selvaraju et al., 2017) is used and visualized to show how the model makes its classification decisions. Specifically, Grad-CAM uses the gradient of the classification score concerning the convolutional features determined by the network to understand which parts of the image are most important for classification. As shown in **Figure 5**, the red area on the Grad-CAM heat map is what the model is focusing on. The results show that in these images where pests are small or easily confused, the region of interest for backbone only includes a small portion of pests, while the model integrating backbone and FFM focuses on most of the body of pests, which means through the method, the shape and category of pests are more accurately identified using fine-grained information.

### 4.5 Ablation study

In this subsection, several ablation experiments are conducted on the test dataset of IP102 to validate the effectiveness of each component of the proposed model.

**Figure 6:**
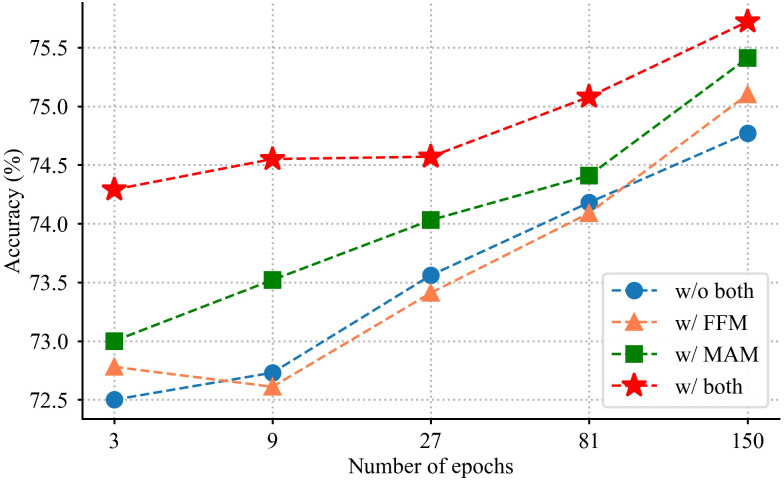
Validation accuracy under different training epochs.

Firstly, the classification performance using the backbone network is validated. It achieves 74.78% accuracy on IP102. Only the backbone network and FFM are combined in the next ablation experiment. The experimental data in **Table 4** shows that the classification accuracy obtained by combining backbone and FFM is 0.32% higher than that of the backbone network. The reason for such a result is that the proposed method is capable of using fine-grained information to distinguish pests. In the third experiment, the effectiveness of MAM is validated. As seen in the fourth row of **Table 4**, the classification results have significantly improved compared to the backbone network, with almost no increase in model parameters. Finally, the full architecture is validated, which achieves the best classification accuracy. **Figure 6** shows the model performance using different combination strategies of the proposed method. It is observed that the learning capacity obtained from the model is significantly more advantageous than others.

**Table 4:** Ablation experiments of different methods on IP102.

Backbone	FFM	MAM	Params (M)	Accuracy (%)
ConvNeXt-L			196.360	74.78
	✓		224.692	75.10
		✓	196.363	75.41
	✓	✓	224.694	75.74

This trade-off between computational complexity and accuracy is a critical consideration when deploying such models in resource-constrained environments. As observed in **Table 4**, the introduction of each module results in incremental changes in both the number of parameters and accuracy. The inclusion of the FFM leads to an increase in the number of parameters, from 196.360M to 224.692M, reflecting the added complexity of this module. Despite this increase, the accuracy improves marginally from 74.78% to 75.10%. This suggests that while FFM introduces additional computational overhead, it enhances the model’s ability to integrate features, resulting in a small but noticeable performance gain. It also indicates that simply increasing the number of parameters does not necessarily lead to a significant improvement in model performance. The introduction of the MAM also results in an increase in parameters, albeit a minimal one (196.360M to 196.363M). Despite the marginal increase in computational complexity, the accuracy improves more substantially, from 74.78% to 75.41%. This highlights the effectiveness of MAM in capturing long-range channel dependencies, contributing significantly to performance improvement without adding a substantial computational burden. When both modules are applied together, the model’s parameter count increases to 224.694M, showing a combined effect on computational complexity. However, the accuracy further improves to 75.74%, indicating that the two modules complement each other, with FFM enhancing feature integration and MAM refining channel-wise attention, leading to a higher overall performance.

### 4.6 Generalization ability

**Table 5** shows the classification performance of different methods on IP102. To further test the generalization ability of the proposed model, another experiment on two insect pest datasets (D0 and Li) is conducted. Before that, the appropriate ratio needs to be chosen for splitting the training and testing sets, as different ratios may have some impact on the experimental results. As shown in **Table 6**, setting 75% of the images as the training set and 25% of the images as the testing set achieved the best classification results compared to the other two methods of dataset partitioning. The proposed method is compared to the state-of-the-art methods on D0 and Li and achieves accuracies of 99.82% and 98.77%, outperforming most methods on these two datasets. **Table 7** shows the classification performance of different methods. The experimental results demonstrate that the proposed model has good generalization ability.

**Table 5:** Classification performance of different methods on IP102.

Method	Backbone	Inference Time (ms)	Acc. (%)	F1. (%)
ViT-B/16 (Dosovitskiy et al., 2020)	Transformer	9.36	72.13	71.81
ViT-L/16 (Dosovitskiy et al., 2020)	Transformer	18.16	69.53	69.01
DeiT-B/16 (Touvron et al., 2021)	Transformer	8.83	71.11	70.72
DeiTV2-B/16 (Touvron et al., 2022)	Transformer	11.13	71.82	71.42
DeiTV2-L/16 (Touvron et al., 2022)	Transformer	21.47	72.64	72.32
SwinT-B (Liu et al., 2021)	Transformer	45.17	74.17	73.96
SwinTV2-B (Liu et al., 2022b)	Transformer	50.31	74.31	73.98
ResNet50 (He et al., 2016)	CNN	11.46	68.50	68.02
ResNet152 (He et al., 2016)	CNN	34.30	69.06	68.39
VGG-16 (Simonyan and Zisserman, 2014)	CNN	2.06	66.44	66.01
ConvNeXt-B (Liu et al., 2022c)	CNN	19.07	74.31	73.97
ConvNeXt-L (Liu et al., 2022c)	CNN	19.34	74.78	74.54
FR-ResNets (Ren et al., 2019)	CNN	–	55.24	54.18
FusionSum (Nanni et al., 2020)	CNN	–	61.44	59.20
GAEnsemble (Ayan et al., 2020)	CNN	–	67.13	67.17
ResNet50+STN+ISAN (Yang et al., 2021)	CNN	–	73.29	–
CNNs+Exp+ExpLR (Nanni et al., 2022)	CNN	62.31	74.11	73.00
CTF (Peng and Wang, 2022)	CNN+Transformer	17.42	74.89	–
AM-ResNet (Zhang et al., 2022)	CNN	–	56.10	–
GAEnsemble (Ayan, 2023)	CNN	–	71.84	64.06
ResNet50+EFLM+AFFM (Chen et al., 2023)	CNN	–	73.90	73.60
Modified-SwinT (Guo et al., 2023)	Transformer	12.21	74.15	60.83
MMALNet+DNVT+ResNet50 (Xia et al., 2023)	CNN+Transformer	–	74.20	67.79
DWViT-ES (Hechen et al., 2024)	Transformer	–	**76.00**	–
Proposed method	CNN+Transformer	21.72	75.74	**75.38**

The bold values represent the optimal values for each column.

**Table 6:** Classification performance using different dataset partitioning methods.

Method	Acc. (%)	
	D0	Li
70% for training and 30% for testing	99.19	97.73
75% for training and 25% for testing	99.82	98.77
80% for training and 20% for testing	99.11	98.04

**Table 7:** Classification performance of different methods on D0 and Li.

Method	D0		Li	
	Acc. (%)	F1. (%)	Acc. (%)	F1. (%)
ViT-B/16 (Dosovitskiy et al., 2020)	99.65	99.65	97.55	97.55
ViT-L/16 (Dosovitskiy et al., 2020)	99.65	99.65	97.82	97.82
DeiT-B/16 (Touvron et al., 2021)	99.65	99.64	98.09	98.09
DeiTV2-B/16 (Touvron et al., 2022)	99.38	99.38	97.96	97.95
DeiTV2-L/16 (Touvron et al., 2022)	99.73	99.73	98.70	98.70
SwinT-B (Liu et al., 2021)	99.73	99.73	98.50	98.50
SwinTV2-B (Liu et al., 2022b)	99.56	99.55	91.21	91.17
ResNet-50 (He et al., 2016)	98.14	98.13	97.21	97.19
ResNet-152 (He et al., 2016)	98.94	98.94	97.55	97.54
VGG-16 (Simonyan and Zisserman, 2014)	98.76	98.75	95.43	95.42
ConvNeXt-B (Liu et al., 2022c)	99.73	99.73	98.36	98.36
ConvNeXt-L (Liu et al., 2022c)	99.65	99.64	98.30	98.29
MLLF + MKB (Xie et al., 2018)	89.30	–	–	–
CNNs (Thenmozhi and Srinivasulu Reddy, 2019)	95.97	–	–	–
GAEnsemble (Ayan et al., 2020)	98.81	98.81	–	–
GoogleNet (Li et al., 2020)	–	–	96.67	–
ResNet50+STN+ISAN (Yang et al., 2021)	–	–	96.78	–
CNNs+Exp+ExpLR (Nanni et al., 2022)	99.81	99.71	–	–
CTF (Peng and Wang, 2022)	99.47	–	97.94	–
GAEnsemble (Ayan, 2023)	**99.89**	**99.86**	–	–
ResNet50+EFLM+AFFM (Chen et al., 2023)	99.80	99.80	–	–
MMALNet+DNVT+ResNet50 (Xia et al., 2023)	99.89	99.85	–	–
Proposed method	99.82	99.82	**98.77**	**98.77**

The bold values represent the optimal values for each column.

## 5 Discussion

### 5.1 Model limitations

Although the proposed method achieves state-of-the-art accuracy, there are still shortcomings, as listed below. Firstly, the issue of imbalanced datasets mentioned earlier (in Section 3.1) has not been resolved yet.

To evaluate the classification performance of the model on data with different sample sizes, IP102 is divided into three groups: The head group includes categories with more than 200 samples, the tail group includes categories with less than 100 samples, and the medium group contains the remaining categories. As shown in **Table 8**, the proposed model performed the best in the medium group but showed a decrease in performance in the head and tail groups because imbalanced data numbers across classes make deep models biased to head classes and perform poorly on tail classes (Zhang et al., 2023). However, the proposed model performed worse in the head group than in the tail group. The possible reason is that the number of samples in the head category of the dataset is too large compared to the number of samples in the tail category. The excessive number of samples in the head category leads to overfitting of the model, while the insufficient number of samples in the tail category leads to underfitting of the model; therefore, how to solve this problem is a new research direction. The most common method is to resample the training set to balance the distribution of sample sizes between different classes (Zhang et al., 2020); another method is to add class weights to the loss function of the model and set higher penalties for the misclassification of minority class samples (Dang et al., 2021).

**Table 8:** Classification performance of the method on data with different sample sizes.

Groups	Sample sizes	Accuracy (%)	F1. (%)
Head	≥ 200	72.87	79.63
Medium	≥ 100*,<* 200	77.29	82.11
Tail	*<* 100	75.79	81.79
Overall	22619	75.74	75.38

Secondly, considering the complexity of the proposed model, it is not easy to deploy it on edge devices. As shown in **Table 2**, the proposed model achieves the best performance at the cost of almost 1 to 10 times more parameters compared to models using other backbone networks. The main reason is that the backbone network used has a large number of parameters. When deploying deep learning models on resource-constrained devices, such as drones or mobile devices, reducing model complexity is crucial. Model compression emerges as an effective solution for this challenge. Model compression aims to reduce the storage and computational requirements of the model while maintaining its performance. Common compression techniques include weight pruning, quantization, and low-rank decomposition, all of which reduce redundant parameters and operations, making the model more suitable for low-power devices. On the other hand, knowledge distillation can be used to reduce the complexity of the model. Knowledge distillation, a model compression technique, trains a smaller “student” model to mimic the behavior of a larger “teacher” model, thereby achieving a balance between performance and efficiency. During the distillation process, the student model learns not only from the hard labels but also from the soft labels (i.e., the probability distributions) produced by the teacher model. This allows the student model to capture more nuanced feature representations and improve its generalization ability. In terms of specific implementation, the model proposed in this article can be used as a teacher model to train a lightweight model such as MobileNet.

### 5.2 Application deployment

**Figure 7:**
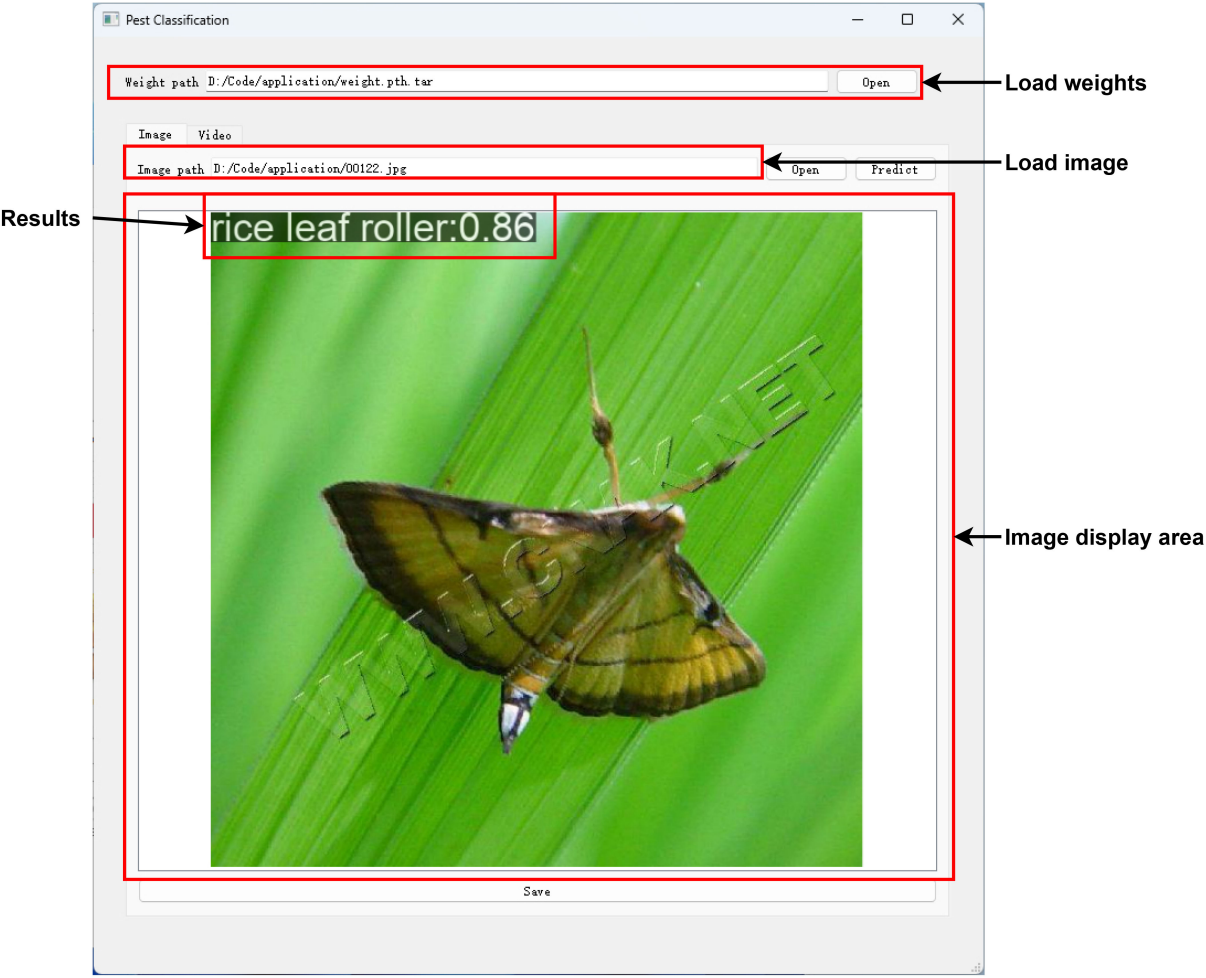
Desktop application.

Due to the limited computing resources and memory of embedded devices, this paper demonstrates an implementation approach to the personal computer. A desktop application called Pest Classification has been developed, which can be used to load trained model weights and identify pests, and **Figure 7** shows the application’s user interface. The pest classification software can be divided into several areas. Model weights and pest images can be customized for loading in the above area. Then, the recognition result, including the pest category name and possibility, will be displayed in the image display area after pressing the predict button. Finally, the result will be saved after pressing the save button. In addition, the developed application supports reading video streams and performing real-time pest identification. In general, the results will be displayed within 0.5 seconds, which can be ignored for image recognition, but the delay is relatively high for real-time pest recognition. Through model lightweight optimization and edge computing technology, efficient device-side inference can be achieved to meet the real-time requirements of different scenarios. At the same time, combined with federated learning and a data closed-loop system, the model performance can be continuously optimized to adapt to regional pest morphological differences. In practical application, this application can improve the accuracy of pest identification, reduce the amount of pesticide use, and provide reliable data support for precision agriculture, which has a wide range of application prospects and promotion value. The source code is available at https://github.com/qianyiheng888/PestClassification/.

### 5.3 Future work

Future work in this domain could focus on evaluating the proposed model on larger and more diverse datasets to further assess its generalization capabilities across a wide range of crop types, pest species, and environmental conditions. Expanding the scope of testing to include datasets with varying levels of image quality, lighting conditions, and seasonal variations would provide valuable insights into the model’s robustness and adaptability in real-world scenarios. Furthermore, the integration of domain-specific knowledge, such as pest behavior patterns and crop growth stages, could enhance the model’s contextual awareness, enabling more accurate and timely pest classification. These future directions could lead to the development of a more comprehensive, reliable, and scalable pest classification system, significantly benefiting precision agriculture and sustainable pest management practices.

## 6 Conclusions

This study presents a novel deep-learning architecture for crop pest classification, designed to address practical challenges in agricultural monitoring and pest management. The architecture, a dual-branch structure that combines Convolutional Neural Networks and Transformer models, aims to improve classification accuracy by integrating fine-grained feature representation, multi-scale feature fusion, and long-range dependency modeling. The key components of this architecture include the Feature Fusion Module (FFM) and the Mixed Attention Module (MAM). The FFM enhances the extraction of detailed features and adaptively selects key information, while the MAM introduces an attention mechanism to capture long-range dependencies within the channel domain. The model’s performance was evaluated across three datasets, IP102, D0, and Li, achieving test accuracies of 75.74%, 99.82%, and 98.77%, respectively. These results highlight the model’s ability to effectively balance fine-grained feature extraction with deep semantic learning, which is critical for accurately identifying pests in real-world agricultural environments. Compared to traditional single-branch networks, the proposed dual-branch architecture prevents the loss of fine-grained features during downsampling and enables better feature expression for small targets, which are often challenging to detect. Additionally, the architecture’s cross-channel information exchange and ability to suppress background noise improve its robustness, particularly in complex environments. By incorporating Transformer blocks, this architecture combines the strengths of CNNs in capturing local patterns and Transformers in modeling global dependencies, achieving superior performance with small computational overhead. The experimental results demonstrate that the model not only achieves state-of-the-art accuracy but also maintains practical inference times, making it suitable for real-time deployment in precision agriculture. The high performance makes the proposed method highly effective for large-scale pest detection, contributing to improved pest management practices and better crop protection strategies in agricultural settings. Moreover, an application is developed to meet the users’ needs for pest identification. In the future, this research aims to explore model lightweighting and integration of multi-domain knowledge to achieve higher accuracy with fewer images and smaller model complexity in practice.

## Data Availability

The original contributions presented in the study are included in the article/**Supplementary Material**, further inquiries can be directed to the corresponding author.
